# Gastrointestinal complications of hepatic glycogen storage disease: a national survey questionnaire study in China

**DOI:** 10.1186/s13023-025-03570-1

**Published:** 2025-01-28

**Authors:** Xiaoyan Zhang, Zexiong Su, Jiaxing Wu, Hanshi Zeng, Xun Jiang, Ying Wang, Huiqing Shen, Xiaoli Xie, Yuan Xiao, Qing Tang, Xiaoping Luo, Xuemei Zhong, Huan Chen, Jiaoli Lan, Yongxin Chen, Xiaolu Zeng, Huiqiong Zhang, Zhiling Li, Yuxin Zhang, Min Yang

**Affiliations:** 1 Department of Pediatrics, Guangdong Provincial People’s Hospital, The Second School of Clinical Medicine, Guangdong Academy of Medical Sciences, Southern Medical University, Guangzhou, 510080 China; 2 Department of Pediatrics, The Second Affiliated Hospital, Air Force Military Medical University, Xi’an, 710038 China; 3 Division of Pediatric Gastroenterology and Nutrition, Xinhua Hospital, Shanghai Jiao Tong University School of Medicine, Shanghai, 200092 China; 4 Department of gastroenterology, Beijing Children’s Hospital, Capital Medical University, National Center for Children’s Health, Beijing, 100045 China; 5 Chengdu Women’s and Children’s Central Hospital, School of Medicine, University of Electronic Science and Technology of China, Chengdu, 610015 China; 6 Department of Pediatrics, Ruijin Hospital, School of Medicine, Shanghai Jiao Tong University, Shanghai, 200025 China; 7 Department of Pediatrics, The First Affiliated hospital of Guangxi Medical University, Nanning, 530021 China; 8 Department of Pediatrics, Tongji Hospital, Tongji Medical College, Huazhong University of Science and Technology, Wuhan, 430030 China; 9 Department of gastroenterology, Capital Institute of Pediatrics, Beijing, 100020 China; 10 Department of gastroenterology, Guangzhou Women and Children’s Medical Center, Guangzhou Medical University, Guangzhou, China; 11 Department of Pediatrics, Guangdong Provincial People’s Hospital, No.106, Zhongshan 2nd Road, Guangzhou, 510080 China

**Keywords:** Hepatic glycogen storage disease, Uncooked corn starch, Gastrointestinal complications, Endoscopy, Inflammatory bowel disease

## Abstract

**Background:**

Hepatic glycogen storage diseases (GSD) are inborn errors of metabolism with abnormal storage or utilization of glycogen, a complex disease with significant genetic heterogeneity and similar clinical manifestations. This study aimed to describe the gastrointestinal symptoms and endoscopic features of hepatic GSD, including types Ia, Ib, III, VI, and IX, to provide evidence for etiology and treatment.

**Methods:**

A national cohort survey questionnaire was distributed to patients diagnosed with GSD type Ia, Ib, III, VI, and IX through genetic testing or their parents in mainland China in May 2022. Expert gastroendoscopists performed endoscopic examinations and evaluations in 10 hospitals. Descriptive statistics were used for analysis.

**Results:**

A total of 315 patients with hepatic GSD were included in the study, including 191 males and 124 females, the median age at diagnosis was 1.42 years (range = 0.08–36.08 years), with a median age of 5.42 years at the time of investigation (range = 0.58–38.08 years). 95% of patients relied on uncooked corn starch (UCCS) for blood glucose maintenance, and the median age of initiation was 18.5 months (range = 4-360 months). The common characteristics of these GSDs were hypoglycemia, lactic acidosis, anemia, liver enlargement, hyperlipidemia, hyperuricemia, and weakness. More than 60% of patients reported gastrointestinal symptom including anorexia, nausea/vomiting, abdominal pain, abdominal bloating, diarrhea, and mucus or bloody stool. The incidence of gastrointestinal symptoms in patients with GSD type Ia, Ib, III, VI and IX were 68%, 68%, 48%, 59% and 48%, respectively. A total of 48 GSD patients underwent gastroenteroscopy, 54% (26/48, 24 patients with type Ib, 1 patient with type Ia and 1 with III) were diagnosed with GSD-associated inflammatory bowel disease (IBD), the endoscopic images showed mucosal edema, erythema, erosions, single or scattered multiple large, deep, round ulcers and strictures, without linear ulcers and cobblestone mucosal lesions.

**Conclusion:**

Patients with GSD type Ia, Ib, III, VI and IX had different degrees of gastrointestinal complications, among which patients with type Ia, Ib and III were the most prominent, and the proportion of GSD-Ib patients had a higher proportion of GSD-associated IBD.

**Supplementary Information:**

The online version contains supplementary material available at 10.1186/s13023-025-03570-1.

## Introduction

Hepatic glycogen storage disease (GSD) is a group of rare congenital disorders caused by the lack of specific enzymes involved in the utilization and storage of glycogen [1, 2], the prevalence of GSD is estimated to range from 2 to 5 cases per 100,000 live births [3]. More than 20 sub-types of GSD exist, the most common type I, including Ia and Ib [3, 4]. Deficiency of the gene encoding glucose-6-phosphatase (*G6PC*) causes GSD type Ia (GSD-Ia), whereas deficiency of the gene encoding glucose-6-phosphate translocase (*G6PT/SLC37A4*) results in GSD type Ib (GSD-Ib) [4]. In most GSD, glycogen catalysis does not take place, leading to improper glycogen storage in the body, particularly in the liver and/or muscles [4]. As a result of defective glycogen metabolism, patients with GSD develop hypoglycemia, hepatomegaly, acidosis, hyperlipidemia, hyperuricemia, failure to thrive, fatigue, and muscle weakness [1, 3, 4].

In the pediatric population, patients with GSD face many unique challenges, the manifestations appear after birth and persist from infancy to adulthood. Children with GSD suffer from anemia, hypoalbuminemia, malnutrition, and failure to thrive. Clinical studies based on case reports or small-scale cohorts showed that patients with GSD primarily seek medical treatment from departments specializing in endocrinology and metabolism or immunology. A minority of patients with GSD-Ib, due to recurrent or persistent gastrointestinal symptoms, were diagnosed with GSD-associated inflammatory bowel disease (IBD) through endoscopic examination, and gradually recognized and understood by gastroenterologists [5–10]. Recently, *SLC37A4* and *G6PC3* have been identified as a monogenic gene associated with IBD in GSD-Ib [11, 12]. Studies suggested that deficiency in G6PC3 was a significant factor in the development of IBD due to neutropenia. However, Dale’s clinical study found that Granulocyte colony-stimulating factor (G-CSF), which increased the number of neutrophils, does not prevent the occurrence and progression of IBD in patients with GSD-Ib [13]. Studies have also reported GSD-associated IBD in patients with GSD-Ia [14, 15] and GSD-III [16], while neutropenia is not present in GSD-Ia and GSD-III. In addition to genetic inheritance, GSD-associated IBD is also related to diet, intestinal microecology, and environmental factors. Therefore, studying the gastrointestinal symptoms and endoscopic features of GSD type I in comparison to other types of hepatic GSD under the same dietary background of uncooked corn starch (UCCS) may provide insights for IBD etiology research.

Recently, we reported gut microbiota imbalances characterized by heterogeneous oral pathogen outgrowth and immature gut microbiota in patients with GSD-Ia and Ib. Macrophages clustered in the colonic mucosa of patients with high expression of CCL4L2 and interacted with colonic epithelial cells and fibroblasts through CCL4L2-VSIR signals to regulate epithelial barrier function [17]. In this study, we present clinical data from a cohort study of 315 patients with 5 sub-types of hepatic GSD, including type Ia, Ib, III, VI, and IX, and 95% of patients who relied on UCCS for blood glucose maintenance. We found digestive symptoms not only in GSD-Ia and GSD-Ib, but also in GSD-III, VI, and IX. We reveal unique endoscopic features of GSD-associated IBD that are more common in GSD-Ib.

## Materials and methods

### Participants

A multi-center cross-sectional questionnaire survey was used in this study, and a total of 10 hospitals participated. The recruited subjects were patients diagnosed with hepatic GSD by genetic testing at tertiary hospitals or children’s specialized hospitals in China. This study was approved by the Medical Ethics Committee of Guangdong Provincial People’s Hospital (ID: 202205301). Consent for the questionnaire study was obtained from all participants or their legal guardians (for participants under 18 years).

### Study design

The questionnaire comprising 26 questions was sent to patients or parents of children aged 0–18 years diagnosed with hepatic GSD. The questionnaire was sent and collected in May 2022, and it encompassed several topics including patient demographics, GSD genetic diagnosis and typing, clinical manifestations, gastrointestinal and infection symptoms, biological and metabolic parameters, endoscopy, diet, and drug treatment. Gastroenterologists and endoscopists evaluated the clinical and endoscopic features of patients with GSD. All data were reassessed and any uncertain responses were cross-verified by the children’s gastroenterologist via telephone.

### Statistical analysis

Descriptive statistics were used in this study. Means (with SDs) and medians (with IQRs) are presented for continuous variables, and numbers or percentages are used for categorical variables. All statistical analyses were performed using R language (version 4.4.1). Chi-squared tests and Fisher’s exact tests are used to compare frequency distributions between different groups to see if there are significant differences. For chi-square tests, if the expected frequency is less than 5, the Fisher test is used instead. The significance level for all statistical tests was set at *p* < 0.05. Descriptive analyses were conducted using the GraphPad Prism software v9.0.2.

## Results

### General information of cohort

This study participants included hepatic GSD from 27 different regions of China, including type Ia, Ib, III, VI, IX, a total of 315 patients. Data from 34 patients were excluded from the study because of non-genetic diagnoses or incomplete information. Table 1 presents the demographic profile of 315 patients with GSD, of whom 191 males (61%) responded to the questionnaire. The median age at initial diagnosis was 1.42 years (range = 0.08-36.08years), whereas the median age at the time of the survey was 5.42 years (range = 0.58-38.08years). The study encompasses five sub-types of hepatic GSD Ia, Ib, III, VI, and IX, the Top 5 nucleotide change alleles, and variant types of different sub-types of GSD shown in Table 2. In this group of patients, according to the number of cases, the types of GSD were: Type Ia (128 patients [41%]), Type Ib (69 patients [22%]), type III (54 patients [17%]), type IX (42 patients, [13%]), and VI (22 patients [7%]). Of note, 7% of patients had at least one family member diagnosed with GSD (Table 1). Common symptoms of the patient with hepatic GSD include hypoglycemia (58%), hepatomegaly (83%), lactic acidosis (18%), weakness (20%), hyperlipidemia (16%), hyperuricemia (18%), renal enlargement (9%) and anemia (12%) (Table 3).

**Table 1:** Demographic profile and medical management of the 315 patients with GSD

Genotypes	total	Ia	Ib	III	VI	IX
Number (n, %) to all	315	128(41%)	69(22%)	54 (17%)	22 (7%)	42 (13%)
Male (n, %)	191(61%)	78(61%)	35(51%)	32 (59%)	11 (50%)	35 (83%)
Female (n, %)	124(39%)	50(39%)	34(49%)	22 (41%)	11 (50%)	7 (17%)
**Age (year**,** y)**						
Median	5.42	5.63	7.42	5.63	4.67	4.83
Range	0.58–38.08	0.67–33.08	0.58–38.08	1.67–25.5	1.42-12	1.25–15.92
**Age at GSD diagnosis (year**,** y)**						
Median	1.42	1.21	0.67	1.5	2.63	2.21
Range	0.08–36.08	0.08–30	0.17–36.08	0.58–18	1–7.83	0.33–6.42
**Family members with same subtypes of GSD**						
Number (n, %) to all	22(7%)	8(6%)	5(7%)	4(7%)	0	5 (12%)
Parents	8	4	0	1	0	3
Siblings	14	4	5	3	0	2
**Dietary management**						
Any UCCS (n, %)	298/315, 95%	125/128, 98%	64/69,93%	51/54,94%	20/22,91%	38/42,90%
Frequncy of UCCS per day (mean ± s.d.)	4.02 ± 1.08	4.39 ± 0.92	4.31 ± 0.97	3.75 ± 0.88	3.10 ± 1.18	3.13 ± 1.0
Daily UCCS: g/kg/d (mean ± s.d.)	6.07 ± 3.38	6.82 ± 3.83	7.15 ± 3.18	4.69 ± 2.22	4.44 ± 2.06	4.50 ± 2.30
standard formula	34/315, 11%	1/128,1%	2/69,3%	13/54, 24%	4/22, 18%	14/42, 33%
lactose-free formula	96/315, 30%	59/128,46%	30/69,43%	1/54, 2%	3/22, 14%	3/42, 7%
polymer formula	15/315, 5%	3/128,2%	11/69,16%	0/54, 0%	0/22, 0%	1/42, 2%
amino acid formula	12/315,4%	5/128,4%	6/69,9%	0/54, 0%	0/22, 0%	1/42, 2%
**Medical management**						
Mesalamine	28/315, 9%	0/128, 0%	27/69,39%	0/54, 0%	0/22, 0%	1/42, 2%
Empagliflozin	18/315, 6%	0/128, 0%	18/69,26%	0/54, 0%	0/22, 0%	0/42, 0%
G-CSF	30/315,10%	0/128,0%	30/69,43%	0/54, 0%	0/22, 0%	0/42, 0%

s.d., standard deviation. UCCS, uncooked cornstarch. G-CSF, granulocyte colony stimulating factor

**Table 2:** TOP 5 variants in patients with GSD type Ia, Ib, III, VI, and IX

Genotypes	Total	Top nucleotide change allele (*n*, %)	Amino acid change allele	Variant type	Variant analysis method
GSD-Ia	128	c.648G > T (48, 38%)	p.Leu216Leu	synonymous	Sequencing
		c.248G > A (15, 12%)	p.Arg83His	missense	Sequencing
		c.310 C > T (7, 5%)	p.Gln104X	nonsense	Sequencing
		c.262delG (6, 5%)	p.Val88fs	frameshift	Sequencing
		c.326G > A (6, 5%)	p.Cys109Tyr	missense	Sequencing
GSD-Ib	69	c.446G > A (19, 28%)	p.Gly149Glu	missense	Sequencing
		c.572 C > T (8, 12%)	p.Pro191Leu	missense	Sequencing
		c.343G > A (4, 6%)	p.Gly115Arg	missense	Sequencing
		c.1179G > A (2, 3%)	p.Trp393X	nonsense	Sequencing
		c.1042_1043del (2, 3%)	p.Leu348Valfs*53	Frameshift, nonsense	Sequencing
GSD-III	54	c.1735 + 1G > T (8, 15%)	NA	splice	Sequencing
		c.3589–3 C > G (3, 6%)	NA	splice	Sequencing
		c.853 C > T (2, 4%)	p.Arg285X	nonsense	Sequencing
		c.2864del (1, 2%)	p.Pro955Leufs*6	Frameshift, nonsense	Sequencing
		c.2856_2878del (1, 2%)	p.Leu952Phefs*9	Frameshift, nonsense	Sequencing
GSD-VI	22	c.2467 C > T (7, 32%)	p.Gln823X	nonsense	Sequencing
		c.698G + A (4, 18%)	p.Gly233Asp	missense	Sequencing
		c.1969 + 1G > A (2, 9%)	NA	splice	Sequencing
		c.1768 + 2T > C (2, 9%)	NA	splice	Sequencing
		c.1768 + 1G > A (2, 9%)	NA	splice	Sequencing
GSD-IX	42	c.749 C> T (3, 7%)	p.Ser250Leu	missense	Sequencing
		c.870_872delCTT (2, 5%)	p.Phe291del	deletion	Sequencing
		c.400_416dup17 (1, 2%)	p.Leu140Argfs*15	Frameshift, nonsense	Sequencing
		c.545G > A (1, 2%)	p.Gly182Glu	missense	Sequencing
		c.1031 A > G (1, 2%)	p.Asp344Gly	missense	Sequencing

**Table 3:** Clinical findings in patients with GSD type Ia, Ib, III, VI and IX

Genotypes	Total	Ia	Ib	III	VI	IX
**Gastrointestinal symptoms (*n***/***N*** %)						
Anorexia	64/315	24/128	22/69	9/54	3/22	6/42
	20%	19%	32%	17%	14%	14%
Abdominal pain	52/315	14/128	21/69	7/54	4/22	6/42
	17%	11%	30%	13%	18%	14%
Abdominal bloating	68/315	26/128	18/69	10/54	6/22	8/42
	22%	20%	26%	19%	27%	19%
Nausea / vomiting	79/315	36/128	20/69	10/54	6/22	7/42
	25%	28%	29%	19%	27%	17%
Diarrhea	97/315	48/128	29/69	10/54	2/22	8/42
	31%	38%	42%	19%	9%	19%
Mucus / bloody stool	34/315	13/128	15/69	3/54	0/22	3/42
	11%	10%	22%	6%	0%	7%
Any GI problems aforementioned	193/315	87/128	47/69	26/54	13/22	20/42
	61%	68%	68%	48%	59%	48%
**Typical GSD features (n/N %)**						
Hypoglycemia	184/315	87/128	38/69	31/54	12/22	16/42
	58%	68%	55%	57%	55%	38%
Hyperlipidemia	49/315	33/128	11/69	2/54	1/22	2/42
	16%	26%	16%	4%	5%	5%
Anemia	39/315	14/128	22/69	1/54	0/22	2/42
	12%	11%	32%	2%	0%	5%
Enlarged kidney	27/315	17/128	9/69	1/54	0/22	0/42
	9%	13%	13%	2%	0%	0%
Hepatomegaly	261/315	107/128	59/69	48/54	16/22	31/42
	83%	84%	86%	89%	73%	74%
Acidosis	57/315	38/128	17/69	2/54	0/22	0/42
(lactic acidosis)	18%	30%	25%	4%	0%	0%
Fatigue and muscle weakness	63/315	13/128	14/69	28/54	1/22	7/42
	20%	10%	20%	52%	5%	17%
Hyperuricemia	56/315	33/128	17/69	3/54	0/22	3/42
	18%	26%	25%	6%	0%	7%
**Infection symptoms (n/N%)**						
Perianal abscess	75/315	27/128	30/69	6/54	2/22	10/42
	24%	21%	43%	11%	9%	24%
Oral ulcer	76/315	12/128	53/69	4/54	2/22	5/42
	24%	9%	77%	7%	9%	12%
Respiratory tract infection	103/315	40/128	28/69	12/54	6/22	17/42
	33%	31%	41%	22%	27%	40%
Otitis media	22/315	5/128	11/69	4/54	0/22	2/42
	7%	4%	16%	7%	0%	5%
Urinary tract infection	19/315	11/128	4/69	4/54	0/22	0/42
	6%	9%	6%	7%	0%	0%
Gastroenteritis	43/315	16/128	17/69	5/54	0/22	5/42
	14%	13%	25%	9%	0%	12%
Skin infection	19/315	5/128	8/69	1/54	2/22	3/42
	6%	4%	12%	2%	9%	7%
Any infection symptoms aforementioned	192/315	72/128	63/69	22/54	9/22	26/42
	61%	56%	91%	41%	41%	62%

### Gastrointestinal symptoms and extraintestinal manifestations

61% of the patients reported at least one gastrointestinal symptom, including diarrhea (31%), nausea/vomiting (25%), abdominal bloating (22%), anorexia (20%), abdominal pain (17%), mucus/bloody stool (11%) (Table 3). Notably, our survey showed that in addition to Ia and Ib, which have been reported to have GSD-associated IBD, gastrointestinal symptoms were also present in patients with other sub-types of GSD, including GSD-III (48%), GSD-VI (59%), and GSD-IX (48%) (Table 3). We employed Chi-squared tests and Fisher’s exact tests to compare the frequency distributions among different groups and discovered that there were significant differences in gastrointestinal symptoms such as abdominal pain (*p* = 0.01439), diarrhea (*p* = 0.001134), and mucus/bloody stool (*p* = 0.01637) among subtypes (Table 4). Then, the pairwise comparisons of gastrointestinal symptoms with *p* < 0.05 were made among subtypes, and found that there were significant differences between Ia and the other four subtypes (Supplementary material for details).

**Table 4:** Chi-square test for gastrointestinal symptoms of patients with GSD type Ia, Ib, III, VI, and IX

Genotypes		Ia	Ib	III	VI	IX	$\:x$_*2*_	p
Gastrointestinal symptoms	(Yes or No)	Number of people						
Anorexia	Yes	24	22	9	3	6	-^*^	0.1288
	No	104	47	45	19	36		
Abdominal pain	Yes	14	21	7	4	6	-^*^	0.01439
	No	114	48	47	18	36		
Abdominal bloating	Yes	26	18	10	6	8	-^*^	0.761
	No	102	51	44	16	34		
Nausea / vomiting	Yes	36	20	10	6	7	4.0676	0.3969
	No	92	49	44	16	35		
Diarrhea	Yes	48	29	10	2	8	18.188	0.001134
	No	80	40	44	20	34		
Mucus / bloody stool	Yes	13	15	3	0	3	-^*^	0.01637
	No	115	54	51	22	39		

Note: ^*^No test statistic is available for Fisher’s exact test. Results that met the conditions of *p* < 0.05 were recognized as significant

In our cohort, a significant proportion of 61% presented with extraintestinal manifestations, including those of respiratory tract infection (33%), oral ulcers (24%), perianal abscess (24%), otitis media (7%), skin infection (6%), and urinary tract infection (6%) (Table 3).

### Laboratory test

Neutropenia is an important feature of GSD-Ib laboratory tests. In this group, a total of 52 patients had neutropenia, among which absolute neutrophil counts between 0.5 × 10^9^/L and 1.5 × 10^9^/L and granulocytopenia (defined as ANC < 0.5 × 10^9^/L) were found respectively in 35 (51%) and 17 patients (25%) (Table 5). Among them, ANC < 0.5 × 10^9^/L was seen not only in GSD-Ib, but also in GSD-Ia (6%), GSD-III (7%), and GSD-IX (17%).

**Table 5:** Biological and metabolic parameters of patients with GSD type Ia, Ib, III, VI, and IX

Genotypes (*n*/*N*, %)	WBC (×10^9^/L)	ANC (×10^9^/L)		Platelet (×10^9^/L)	Hemoglobin (g/L)				Blood glucose (mmol/L)	Lactate (mmol/L)	Uric acid (mmol/L)	Triglyceride (mmol/L)
	< 4.0	0.5 ≤ ANC < 1.5	ANC < 0.5	> 300	> 120	90–120	60–90	< 60	< 2.8	> 1.8	> 420	> 1.7
**GSD-Ia**	3/128 2%	11/128 9%	8/128 6%	99/128 77%	56/128 44%	66/128 52%	3/128 2%	3/128 2%	5/128 4%	112/128 88%	71/128 55%	101/128 79%
**GSD-Ib**	25/69 36%	35/69 51%	17/69 25%	51/69 74%	14/69 20%	44/69 64%	11/69 16%	0/69 0%	2/69 3%	65/69 94%	26/69 38%	47/69 68%
**GSD-III**	9/54 17%	0/54 0%	4/54 7%	32/54 59%	46/54 85%	8/54 15%	0/54 0%	0/54 0%	5/54 9%	27/54 50%	17/54 31%	32/54 59%
**GSD-VI**	0/22 0.0%	0/22 0.0%	0/22 0.0%	13/22 59.09%	16/22 72.73%	6/22 27.27%	0/22 0.0%	0/22 0.0%	0/2 0.0%	9/22 40.91%	3/22 14%	4/22 18%
**GSD-IX**	0/42 0%	0/42 0%	7/42 17%	28/42 67%	24/42 57%	18/42 43%	0/42 0%	0/42 0%	3/42 7%	8/42 19%	5/42 12%	7/42 17%

*WBC, white blood cell. ANC, absolute neutrophil count.

12% of patients with a white blood cell count < 4.0 × 10^9^/L. In addition, 4% of the patients met the criteria of having mild anemia (defined by having a hemoglobin level between 60 and 90 g/L), while 3 patients with GSD-Ia presented with severe anemia (defined by having a hemoglobin level < 60 g/L). High platelet count (platelet count > 300 × 10^9^/L) was observed in patients with all the GSD subtypes, including GSD-Ia (77%), GSD-Ib (74%), GSD-III (59%), GSD-VI (59%), and GSD-IX (67%). The biological and metabolic parameters for patients with GSD are presented in Table 5.

### Endoscopic and histological characteristics of GSDs

**Fig. 1:**
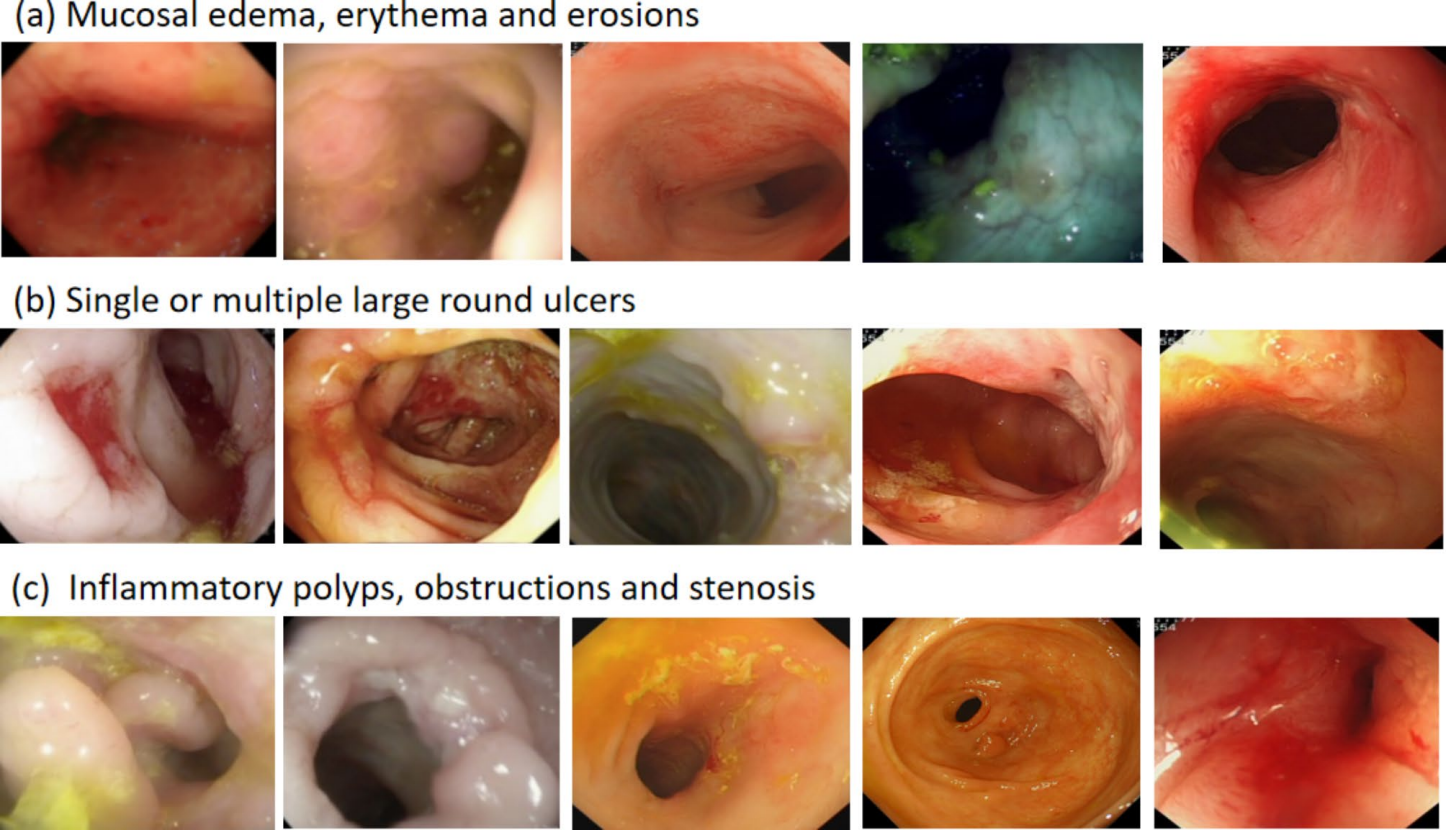
Endoscopic features in pediatric patients with GSD-associated IBD The endoscopic features of GSD-associated IBD in pediatric patients. (**a**) mucosal edema, erythema and erosions. (**b**) single or multiple large round ulcers. (**c**) inflammatory polyps, obstructions and stenosis

**Fig. 2:**
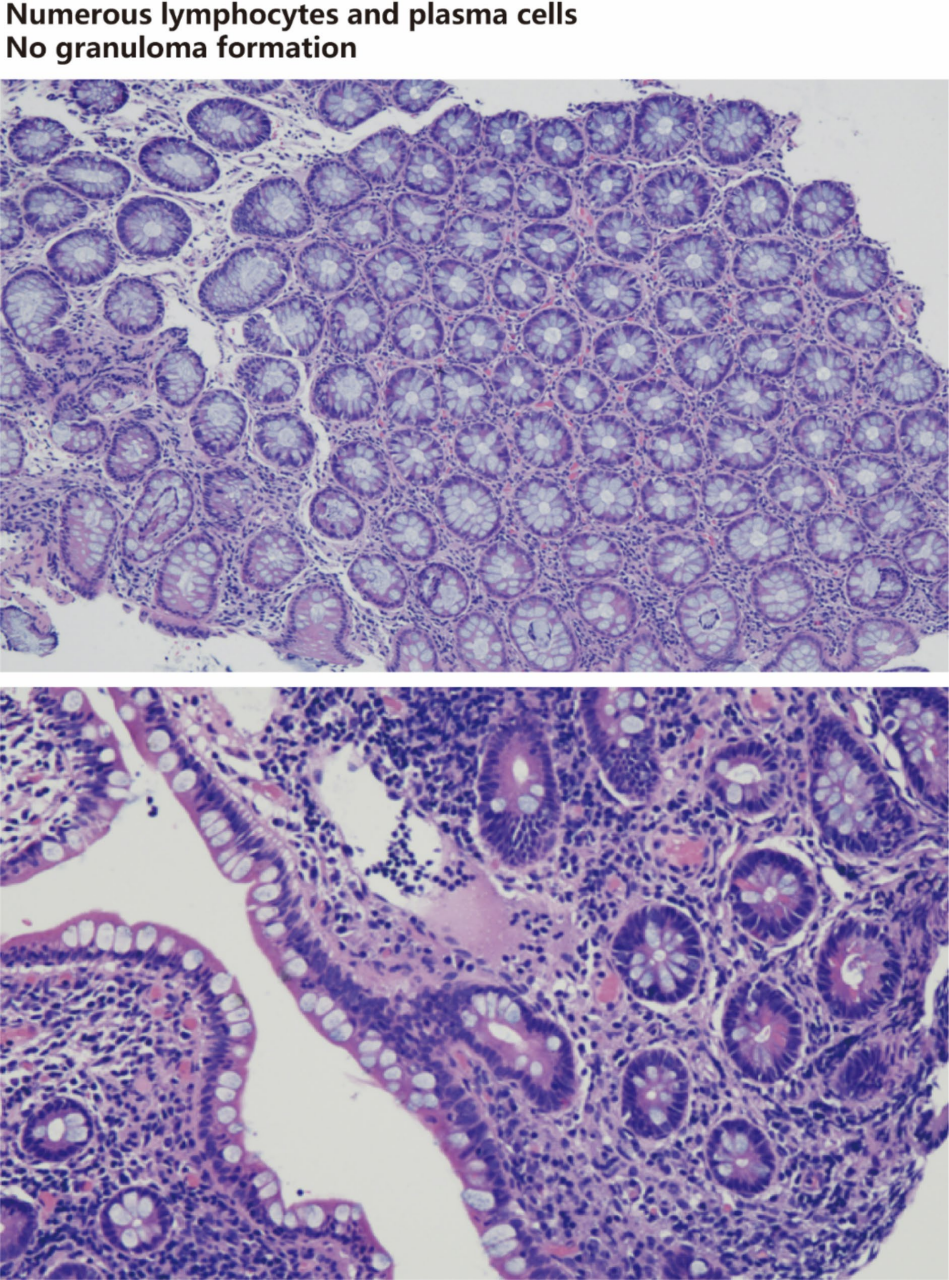
Histology of colonic mucosa from patients with GSD-associated IBD Histological features of colonic mucosa in patients with GSD-associated IBD. There were abundant lymphocytes and plasma cells in the lamina propria of colon mucosa, scattered infiltration of eosinophils, no neutrophil infiltration and granuloma formation

In this group, 48 patients underwent gastroenteroscopy, and detailed endoscopic reports were provided (Table 6). Of the 12 patients with GSD-Ia, 2 reported colitis (17%) and 1 reported GSD-associated IBD (8%). All 28 patients with GSD-Ib had abnormal gastrointestinal endoscopic images including mucosal edema, erythema, erosion, single or multiple deep large round ulcers, inflammatory polyps, obstruction, and stenosis. GSD-associated IBD was reported in 24 patients (86%), of which 14 patients (50%) reported colonic stenosis, 1 patient reported colonic perforation (4%), and 4 patients reported colitis (14%). Of the 5 patients with GSD-III, 1 reported IBD (20%) (not shown in endoscopic images). The endoscopic images showed no abnormalities in 2 patients with GSD-VI and 1 patient with GSD-IX. Typical endoscopic images of GSD-associated IBD were shown in Fig. 1, including deep round ulcers, strictures, and obstruction, with no longitudinal ulcers or cobblestone-like mucosal lesions, similar to our previously reported [17, 18]. Histological examination of colonic mucosa revealed lymphocytic infiltration in the lamina propria, with abundant lymphocytes and plasma cells in ascending, transverse, descending, and sigmoid colon, and scattered eosinophilic infiltration without neutrophil infiltration or granuloma formation (Fig. 2).

**Table 6:** Endoscopic and pathological findings of 48 patients undergoing gastroenteroscopy

Genotypes	Total	Ia	Ib	III	VI	IX					
**Endoscopic features (*n***/***N*** %)											
Mucosal edema, erythema	32/48	3/12	28/28	1/5	0/2	0/1					
	67%	25%	100%	20%	0%	0%					
Erosion	7/48	2/12	4/28	1/5	0/2	0/1					
	15%	17%	14%	20%	0%	0%					
Ulcers	19/48	1/12	17/28	1/5	0/2	0/1					
	40%	8%	61%	20%	0%	0%					
Inflammatory polyps	3/48	0/12	3/28	0/5	0/2	0/1					
	6%	0%	11%	0%	0%	0%					
Stenosis	14/48	0/12	14/28	0/5	0/2	0/1					
	29%	0%	50%	0%	0%	0%					
Perforation	1/48	0/12	1/28	0/5	0/2	0/1					
	2%	0%	4%	0%	0%	0%					
**Pathological features (n/N %)**											0%
Mild chronic inflammation	26/48	2/12	20/28	1/5	2/2	1/1					
	54%	17%	71%	20%	100%	100%					
Moderate chronic inflammation	7/48	1/12	6/28	0/5	0/2	0/1					
	15%	8%	21%	0%	0%	0%					
Severe chronic inflammation	2/48	0/12	2/28	0/5	0/2	0/1					
	4%	0%	7%	0%	0%	0%					
Lymphocytic infiltration	30/48	3/12	26/28	1/5	0/2	0/1					
	63%	25%	93%	20%	0%	0%					
Eosinophilic infiltration	2/48	0/12	2/28	0/5	0/2	0/1					
	4%	0%	7%	0%	0%	0%					
Glandular	2/48	0/12	2/28	0/5	0/2	0/1					
ectasia	4%	0%	4%	0%	0%	0%					
**Endoscopic diagnosis (n/N %)**											
GSD-associated IBD	26/48	1/12	24/28	1/5	0/2	0/1					
	54%	17%	71%	20%	0%	0%					
Colitis	6/48	2/12	4/28	0/5	0/2	0/1					
	13%	17%	14%	0%	0%	0%					
Colonic stenosis	14/48	0/12	14/28	0/5	0/2	0/1					
	29%	0%	50%	0%	0%	0%					
Colonic perforation	1/48	0/12	1/28	0/5	0/2	0/1					
	2%	0%	4%	0%	0%	0%					

### Dietary and medical management

Dietary management remains the primary treatment for patients with hepatic GSD. In our cohort, 95% of patients consumed UCCS (Table 1) 4 times per day at a mean dose of 6.07 g/kg /day. The median age at initiation of the UCCS diet was 18.5 months. In addition to UCCS, these include lactose-free formula (30%), standard formula (11%), polymer formula (5%), and amino acid formula (4%).

Granulocyte-colony stimulating factor (G-CSF) is recommended for neutropenia in patients with GSD-Ib. In this group, 30 patients with GSD-Ib received G-CSF. However, a colonoscopy of 10 patients treated with G-CSF still showed mucosal lesions and diagnosed IBD. In addition, some patients with GSD-Ib have received mesalamine (39%) or empagliflozin (26%). The dietary and medical management for patients with GSD was summarized in Table 1.

## Discussion

Glycogen storage diseases are rare genetic disorders that impact various physiological systems. The incidence of GSD is estimated to be 1 case in 20,000–43,000 live births [3]. With the development of genetic diagnostics, GSD can be diagnosed at an early age. In this study, we demonstrated the prevalence of digestive symptoms in patients with different subtypes of hepatic GSD (GSD-Ia, Ib, III, VI, and IX) in a large national population in China. To our knowledge, this is the first large national epidemiological study of GSD in China. The digestive symptoms were previously described in patients with GSD-Ib in a French and North American study [9, 10]. In our study, in addition to GSD-Ia and Ib, we included patients with GSD-III, VI, and IX, the 5 types of GSD patients had different degrees of gastrointestinal symptoms at the time of inclusion in this study, and the incidence ranged from 48 to 68%. Common gastrointestinal symptoms included diarrhea, nausea/vomiting, abdominal bloating, anorexia, abdominal pain, and mucus/bloody stool. The gastrointestinal symptoms of patients with GSD-associated IBD are persistent, which may be related to the pathogenesis of IBD as a comorbidities. There 95% of the patients relied on UCCS to maintain blood sugar levels this group, consumed UCCS at a dose of 6.07 g/kg/day (range 4.44–7.15 g/kg/day) at enrolment, with some parents complaining of diarrhea and abdominal pain after UCCS. Whether UCCS is one of the causes of digestive symptoms and IBD needs further study.

At present, the precise cause of numerous gastrointestinal symptoms remains unknown. One hypothesis posits that metabolic issues might have modified the composition of the gut microbiome, thereby triggering these gastrointestinal symptoms. We previously reported that there are distinctions in the gut microbiota composition among individuals with different types of glycogen storage diseases. Further analysis revealed that the intake of raw corn starch had an impact on the gut microbiota, although this effect was less significant than that of genotype, family factors (compared by family pairing), and the region of residence [17].

In this group, the incidence of perianal lesions and recurrent oral ulcers was 24%, respectively, with the highest proportion of type Ib (43% and 77%, respectively), followed by GSD-IX and Ia. It is well known that perianal lesions and recurrent oral ulcers are common parenteral symptoms of IBD and one of the indications of endoscopy. Patients with GSD-associated IBD and colitis exhibit image features, including colonic mucosal edema, erythema, erosion, single or multiple deep large circular ulcers, inflammatory polyps, obstruction, and stenosis [18]. Importantly, these features differ from those typically observed in patients with IBD, characterized by longitudinal ulceration and cobblestone mucosa damage [19]. We found that endoscopic abnormalities (GSD-associated IBD and colitis) were found only in GSD-Ia, Ib, and III, but not in patients with GSD-VI and IX, although they also had gastrointestinal symptoms and consumed UCCS. The pathogenesis of GSD-associated IBD remains unclear.

After extensive investigation, we have to concede that gastrointestinal lesions constitute one of the omissions and challenges in the diagnosis and treatment of children with GSD. Given that clinical managers often neglect the digestive tract status of children with GSD, along with the complex factors such as low blood sugar and the frequent occurrence of such conditions in these children after fasting, there are numerous limitations and difficulties in the clinical implementation of gastroenteroscopy, which further impedes the accurate diagnosis and effective treatment of gastrointestinal lesions in children with GSD. Therefore, we strongly advocate that for children with GSD presenting gastrointestinal symptoms, gastroenteroscopy should be more actively pursued, as it will significantly assist us in diagnosing and treating their gastrointestinal lesions more comprehensively and precisely.

Neutropenia or neutrophil dysfunction has been associated with digestive symptoms in patients with GSD-Ib [6, 9, 10]. Neutrophil defects lead to recurrent infections, which contribute to IBD. In this group, 74% of patients with GSD-Ib had neutropenia, it was also present in 7–17% of children with the other three subtypes, GSD-Ia, GSD-III, and GSD-IX. The cause of neutropenia in these subtypes is unclear.

Our previous study showed that platelets were activated at colonic mucosae in pediatric colitis or IBD [20], consistent with what is observed in adult IBD [21]. Elevated platelet count has been reported in patients with GSD-Ib [22, 23]. In this study, 71% of the patients reported high platelet counts, including those with GSD-Ia (77%), GSD-Ib (74%), GSD-III (59%), GSD-VI (59%), and GSD-IX (67%). Thus, elevated platelet counts may be a common feature of inflammatory digestive complications. Targeting platelet count might be a strategy for managing GSD-associated IBD.

G-CSF, a neutrophil-enhancing growth factor, improved the infectious and digestive symptoms of GSD-Ib [9]. Nevertheless, we found that 10 patients with GSD-Ib were diagnosed with GSD-associated IBD after receiving G-CSF therapy, suggesting that G-CSF administration could not prevent and treat GSD-associated IBD. Recent studies have shown that empagliflozin, a glucose cotransporter sodium-glucose cotransporter 2 inhibitor, is beneficial for treating neutropenia and neutrophil dysfunction in patients with GSD-Ib [24–26]. Recently, we reported the efficacy of empagliflozin in patients with GSD-Ib diagnosed with GSD-associated IBD by clinical presentation and PCDAI score, as well as endoscopic and histological evaluation [18]. In this cohort, 18 patients with GSD-Ib who received empagliflozin showed significant improvement in their gastrointestinal symptoms. However, the potential mechanism of action of empagliflozin requires further study.

This study summarizes the clinical characteristics of patients with GSD-Ia, Ib, III, VI, and IX from mainland China, all five types of GSD have varying degrees of gastrointestinal symptoms. GSD-associated IBD is more common in GSD-Ib, rare in types Ia and III, and not found in types VI and IX.

## Electronic Supplementary Material

Below is the link to the electronic supplementary material.

Supplementary Material 1

## Data Availability

The data reported in this article will be made available for noncommercial, academic purposes upon request.

## Abbreviations

GSD: Glycogen storage disease
UCCS: Uncooked corn starch
IBD: Inflammatory bowel disease
G6PC: Glucose-6-phosphatase
G6PT/SLC37A4: Glucose-6-phosphate translocase
GSD-Ia: GSD type Ia
GSD-Ib: GSD type Ib
G-CSF: Granulocyte colony-stimulating factor
