# Supplementary material for: Gastrointestinal complications of hepatic glycogen storage disease: a national survey questionnaire study in China
Source: Orphanet J Rare Dis. 2025 Jan 28;20:41. doi: 10.1186/s13023-025-03570-1 (PMC11773708; doi:10.1186/s13023-025-03570-1)
Supplement: Supplementary file 1 — Supplementary Material 1 [file 13023_2025_3570_MOESM1_ESM.docx]

Supplementary material

The pairwise comparisons of gastrointestinal symptoms with p<0.05 among subtypes

| **Pairwise comparisons** | | **Gastrointestinal symptoms** | | | | | |
| --- | --- | --- | --- | --- | --- | --- | --- |
|  |  | **Abdominal pain** | | **Diarrhea** | | **Mucus / bloody stool** | |
| **Group1** | **Group2** | **p_value** | **p_adjusted** | **p_value** | **p_adjusted** | **p_value** | **p_adjusted** |
| **Ia** | **Ib** | 5.41E-07 | 5.41E-06 | 5.41E-07 | 5.41E-06 | 5.41E-07 | 5.41E-06 |
| **Ia** | **III** | 8.91E-11 | 8.91E-10 | 8.91E-11 | 8.91E-10 | 8.91E-11 | 8.91E-10 |
| **Ia** | **VI** | 1.82E-24 | 1.82E-23 | 1.82E-24 | 1.82E-23 | 1.82E-24 | 1.82E-23 |
| **Ia** | **IX** | 8.41E-15 | 8.41E-14 | 8.41E-15 | 8.41E-14 | 8.41E-15 | 8.41E-14 |
| **Ib** | **III** | 1.59E-01 | 1.00E+00 | 1.59E-01 | 1.00E+00 | 1.59E-01 | 1.00E+00 |
| **Ib** | **VI** | 1.07E-07 | 1.07E-06 | 1.07E-07 | 1.07E-06 | 1.07E-07 | 1.07E-06 |
| **Ib** | **IX** | 6.37E-03 | 6.37E-02 | 6.37E-03 | 6.37E-02 | 6.37E-03 | 6.37E-02 |
| **III** | **VI** | 1.24E-04 | 1.24E-03 | 1.24E-04 | 1.24E-03 | 1.24E-04 | 1.24E-03 |
| **III** | **IX** | 2.23E-01 | 1.00E+00 | 2.23E-01 | 1.00E+00 | 2.23E-01 | 1.00E+00 |
| **VI** | **IX** | 1.17E-02 | 1.17E-01 | 1.17E-02 | 1.17E-01 | 1.17E-02 | 1.17E-01 |
